# Short-Term Epileptiform Activity Potentiates Excitatory Synapses but Does Not Affect Intrinsic Membrane Properties of Pyramidal Neurons in the Rat Hippocampus In Vitro

**DOI:** 10.3390/biomedicines9101374

**Published:** 2021-10-01

**Authors:** Julia L. Ergina, Dmitry V. Amakhin, Tatyana Y. Postnikova, Elena B. Soboleva, Aleksey V. Zaitsev

**Affiliations:** Sechenov Institute of Evolutionary Physiology and Biochemistry of RAS, 44, Toreza Prospekt, 194223 Saint Petersburg, Russia; for.mail.ergin@gmail.com (J.L.E.); dmitry.amakhin@gmail.com (D.V.A.); tapost2@mail.ru (T.Y.P.); soboleva.elena.1707@gmail.com (E.B.S.)

**Keywords:** temporal lobe epilepsy, hippocampus, 4-aminopyridine, epilepsy model, long-term potentiation, AMPA receptor

## Abstract

Even brief epileptic seizures can lead to activity-dependent structural remodeling of neural circuitry. Animal models show that the functional plasticity of synapses and changes in the intrinsic excitability of neurons can be crucial for epileptogenesis. However, the exact mechanisms underlying epileptogenesis remain unclear. We induced epileptiform activity in rat hippocampal slices for 15 min using a 4-aminopyridine (4-AP) in vitro model and observed hippocampal hyperexcitability for at least 1 h. We tested several possible mechanisms of this hyperexcitability, including changes in intrinsic membrane properties of neurons and presynaptic and postsynaptic alterations. Neither input resistance nor other essential biophysical properties of hippocampal CA1 pyramidal neurons were affected by epileptiform activity. The glutamate release probability also remained unchanged, as the frequency of miniature EPSCs and the paired amplitude ratio of evoked responses did not change after epileptiform activity. However, we found an increase in the AMPA/NMDA ratio, suggesting alterations in the properties of postsynaptic glutamatergic receptors. Thus, the increase in excitability of hippocampal neural networks is realized through postsynaptic mechanisms. In contrast, the intrinsic membrane properties of neurons and the probability of glutamate release from presynaptic terminals are not affected in a 4-AP model.

## 1. Introduction

A significant number of cases of temporal lobe epilepsy in humans develop in healthy people as a result of injury or disease. Acquired epilepsy is often a progressive disease that is resistant to pharmacological treatment [1]. Therefore, it is crucial to know the initial molecular and cellular abnormalities specific to epileptogenesis. Based on this knowledge, more promising therapeutic strategies for the prevention of acquired temporal lobe epilepsy can be developed [2,3].

In vitro brain tissue preparations allow the simple and accessible study of brain networks and provide an opportunity to understand the brain’s molecular and cellular mechanisms of functioning in health and disease with detail that is unattainable in vivo. Therefore, in vitro brain slices are generally recognized as an optimal model for studying epileptiform activity in the brain tissue [4]. Among multiple in vitro models, many researchers utilized a 4-aminopyridine (4-AP)-based model to successfully induce epileptiform activity in the hippocampus and cortical areas [5,6,7,8]. 4-AP blocks voltage-gated potassium channels Kv1.1, Kv1.2, and Kv1.4, which are particularly important for action potential repolarization. This, in turn, promotes the enhanced release of glutamate and, therefore, an overactivation of glutamate receptors [9]. At the same time, GABA-mediated transmission paradoxically facilitates neuronal hyperexcitation in 4-AP-based epilepsy models [8]. Ultimately, both pyramidal neurons and interneurons seem to contribute to the generation of 4-AP-induced epileptiform activity [10,11,12]. 

Even relatively short seizures can lead to activity-dependent structural remodeling of neural circuits, resulting in increased network excitability. Although most mesial temporal lobe structures are highly susceptible to seizures, the hippocampal area demonstrates the heaviest damage in response to seizure activity [13,14]. Several mechanisms can provoke changes in the excitability of neuronal networks, including changes in intrinsic neuronal excitability [15,16,17], potentiation of excitatory synaptic contacts [9,18,19,20,21], changes in synaptic inhibition [22,23,24,25], and cell loss and sprouting of axons [26,27,28]. However, relatively little is known about the precise mechanisms of the network excitability increase resulting from a brief episode of epileptic activity—what specific changes occur at presynaptic and postsynaptic levels, and how these changes affect hippocampal circuit functioning. 

In the present study, using a 4-aminopyridine model of epileptiform activity in vitro, we experimentally investigated mechanisms involved in the increased neuronal excitability in the CA1 hippocampal area. We focused on the alternations that persist 1 h after the short-term epileptiform activity.

## 2. Materials and Methods

### 2.1. Animals and Brain Slice Preparation

Juvenile Wistar rats (postnatal days 21–23) were used in this study. All the experiments were carried out according to the Guidelines on the Treatment of Laboratory Animals effective at the Sechenov Institute of Evolutionary Physiology and Biochemistry of the Russian Academy of Sciences. These guidelines comply with Russian and international standards.

Acute brain slices were obtained as previously described [29]. In brief, rats were decapitated and the brains were quickly removed and placed in ice-cold oxygenated (95% O_2_: 5% CO_2_) artificial cerebrospinal fluid (ACSF) containing (in mM) 126 NaCl, 24 NaHCO_3_, 2.5 KCl, 2 CaCl_2_, 1.25 NaH_2_PO_4_, 1 MgSO_4_, and 10 dextrose. Horizontal entorhinal-hippocampal brain slices (300–350 μm) were prepared with Microm HM 650V vibratome (Microm, Dreieich, Germany) and allowed to recover for 1 h before electrophysiological experiments began. 

### 2.2. Induction of Short-Term Epileptiform Activity In Vitro

Epileptiform activity was induced by an epileptogenic low-magnesium solution with the voltage-gated potassium ion channel inhibitor 4-AP. The solution contained the following (in mM): 120 NaCl, 8.5 KCl, 1.25 NaH_2_PO_4_, 0.25 MgSO_4_, 2 CaCl_2_, 24 NaHCO_3_, 10 dextrose, and 0.05 4-AP. This solution induced epileptiform activity in the slice with a delay of 3–5 min. The brain slices were kept in this solution for 20 min at 30 °C. After that, the slices were washed in ACSF for 1 h. All solutions were oxygenated (95% O_2_/5% CO_2_).

### 2.3. Field Excitatory Postsynaptic Potential (fEPSP) Recordings

Field EPSPs were registered from the CA1 stratum radiatum using a glass microelectrode (0.2–1.0 MΩ) filled with ACSF. Synaptic responses were evoked by local extracellular stimulation of the Schaffer collaterals using a bipolar twisted stimulating electrode made of insulated nichrome wire (0.7 mm in diameter). The stimulating electrode was placed in the stratum radiatum at the CA1–CA2 border at 1 mm from the recording electrode. The dependence of fEPSP amplitude and the fiber volleys (FVs) amplitude on stimulation strength was determined by increasing the current intensity from 25 to 300 μA with a step of 25 μA via an A365 stimulus isolator (World Precision Instruments, Sarasota, FL, USA). Responses were recorded with the Model 1800 Microelectrode AC Amplifier (A-M Systems, Carlsborg, WA, USA). They were digitized with ADC/DAC NI USB-6211 (National Instruments, Austin, TX, USA) using WinWCP v5 software (University of Strathclyde, Glasgow, UK). As previously described [17], the maximum rise slope of the input–output (I/O) relationships (fEPSP amplitude vs. FV amplitude) was calculated for every slice by fitting with a sigmoidal Gompertz function:

(1) $$f\left[I;a,k,I_{infl}\right]=ae^{-e^{\left(-k\left(I-I_{infl}\right)\right)}}$$

where *e* is Euler’s number (e = 2.71828…), *a* is the asymptote of the maximum response amplitude, $I_{infl}$ is the inflection current (in pA), which was the value of the stimulation current at which the maximum slope of the curve was observed, and *k* is the positive number that determines the slope of the curve. The maximum slope of the curve (in Hz/pA) was calculated as a × k/e.

### 2.4. Patch-Clamp Experiments

CA1 hippocampal pyramidal neurons were identified using a Zeiss Axioscop 2 microscope (Zeiss; Oberkochen, Germany) equipped with differential interference contrast optics and the video camera PointGrey Grasshopper3 GS3-U3-23S6M-C (FLIR Integrated Imaging Solutions Inc., Wilsonville, OR, USA). Signals were recorded using a Multiclamp 700B (Molecular Devices, Sunnyvale, CA, USA) patch-clamp amplifier and an NI USB-6343 A/D converter (National Instruments, Austin, TX, USA) using WinWCP 5 software (University of Strathclyde, Glasgow, UK). 

Patch pipettes with tip resistance 2–5 MΩ were pulled from borosilicate filamented glass capillaries (World Precision Instruments, Sarasota, FL, USA) using a P-1000 Micropipette Puller (Sutter Instrument; Novato, CA, USA). The intracellular patch pipette solution for whole-cell recordings contained (in mM) 136 K-Gluconate, 10 NaCl, 5 EGTA, 10 HEPES, 4 ATP-Mg, and 0.3 GTP; pH was adjusted to 7.25 with KOH. A cesium-methanesulfonate-based intracellular patch pipette solution was used for the recordings of the AMPAR- and NMDAR-mediated currents; the composition, in mM, was 127 CsMeSO_4_, 10 NaCl, 5 EGTA, 10 HEPES, 6 QX314, 4 ATP-Mg, and 0.3 GTP; pH was adjusted to 7.25 with CsOH. Access resistance was typically 10–15 MΩ and remained stable during the experiments (< 30% increase) for all cells included in the analysis.

The synaptic responses were evoked with a bipolar stimulating electrode placed at 100–200 μm from the recorded neuron. To evaluate the dependence of the AMPAR-mediated response amplitude from the stimulation current, the AMPAR-mediated EPSCs were recorded in the presence of an NMDAR channel blocker MK-801 (10 µM, Alomone Labs, Jerusalem, Israel) at the holding potential of −56 mV, which is equal to the reversal potential of GABAaR-mediated currents as it was found in our previous studies utilizing the same pipette and extracellular solutions [12,21,30].

The dependence of the evoked EPSC (eEPSC) amplitude on stimulation strength was determined by increasing the current intensity from 0 to 1000 µA via an A365 stimulus isolator (WPI Inc., Blacksburg, VA, USA). The obtained dependence was fitted with a sigmoid Gompertz function (Equation (1)).

In order to investigate the AMPA/NMDA ratio, the AMPAR-mediated EPSCs were recorded at the holding potential of −80 mV in the presence of bicuculline (20 μM), a GABAa receptor blocker. NMDAR-mediated EPSCs were recorded at +40 mV, in the presence of bicuculline and DNQX (10 μM, Tocris Bioscience, Bristol, UK), an AMPAR antagonist. The AMPA/NMDA ratio was calculated as a peak amplitudes ratio. Data were analyzed with Clampfit 10.0 software (Molecular Devices, Sunnyvale, CA, USA). 

Recordings of miniature EPSCs (mEPSCs) were done in the presence of tetrodotoxin (TTX, 0.5 μM; Alomone Labs) and GABAR blockers (picrotoxin, 50 μM and bicuculline, 10 μM, Tocris Bioscience). Miniature events were detected and analyzed using Clampfit 10 software (Molecular Devices, Sunnyvale, CA, USA). The mEPSC amplitudes were determined from the baseline to the peak. 

Intrinsic membrane properties of neurons were evaluated from the voltage responses to the series of 1500-ms current steps with 10–20 pA increments using custom scripts written in Wolfram Mathematica 10 (Wolfram Research, Champaign, IL, USA). Only neurons with the typical regular-spiking pattern were included. 

The resting membrane potential (V_rest_, in mV) was measured as an averaged potential before the current step application. The input resistance (R_input_; in MΩ) was calculated as the voltage–current (V–I ) curve slope. The membrane time constant (τ_m_; in ms) was estimated by fitting a single exponential function to the voltage transient induced by the −25 pA current step. 

The firing rate–current (f/I) curves were used to describe the firing properties of neurons. The firing rate was estimated as the number of action potentials per current step. The rising part of the f/I curve was fitted with a sigmoidal Gompertz function (Equation (1)).

### 2.5. Data Analysis and Statistics

The data were processed with Statistics 8 (StatSoft Inc., Tulsa, OK, USA), OriginPro 8 (OriginLab Corporation, Northampton, MA, USA), and Sigmaplot 12.5 (Systat Software Inc., San Jose, CA, USA). Statistical significance was assessed using the Student’s *t*-test and ANOVA as stated in the text. All data are presented as the mean with the standard error of the mean. *p* < 0.05 was considered statistically significant.

## 3. Results

### 3.1. Epileptiform Activity in Entorhinal-Hippocampal Slices

**Figure 1:**
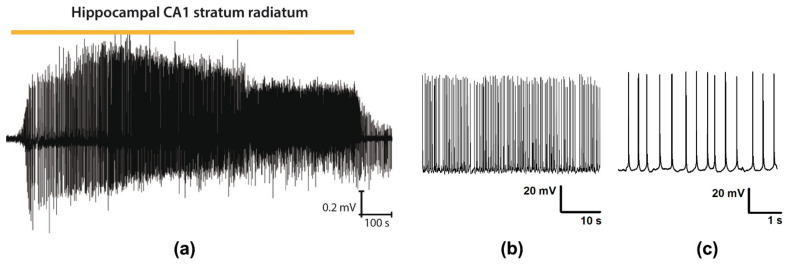
Epileptiform activity in rat brain slices. (**a**) Representative recording of epileptiform activity in the CA1 hippocampal area induced by the epileptogenic solution (local field potential (LFP) recording). (**b**,**c**) The interictal discharges registered in CA1 pyramidal cells (whole-cell current-clamp recordings at different time scale).

This study investigated the short-term (within 1 h) effects of epileptiform activity on synaptic and nonsynaptic plasticity in the hippocampus. Epileptiform activity in rat entorhinal-hippocampal slices was induced by 20-min exposure to the 4-AP-containing bath solution with altered extracellular ion concentrations (8.5 mM K^+^; 0.25 mM Mg^2+^). As we have shown previously, this epileptogenic solution reliably induced discharges in the rat entorhinal cortex approximately 7−10 min after application [30]. In the CA1 hippocampal area, the first discharges emerged even earlier, in 3–5 min (Figure 1). Thus, the total duration of epileptiform activity in the hippocampal network was about 15 min.

### 3.2. Epileptiform Activity Increases the Gain of Input–Output Relationship in CA3-CA1 Synapses 

One hour after washing the sections in Ringer’s solution, we examined the properties of synaptic transmission in the hippocampal CA3-CA1 synapses. This time interval is sufficient to trigger intracellular signaling cascades and induce plasticity [31]. 

**Figure 2:**
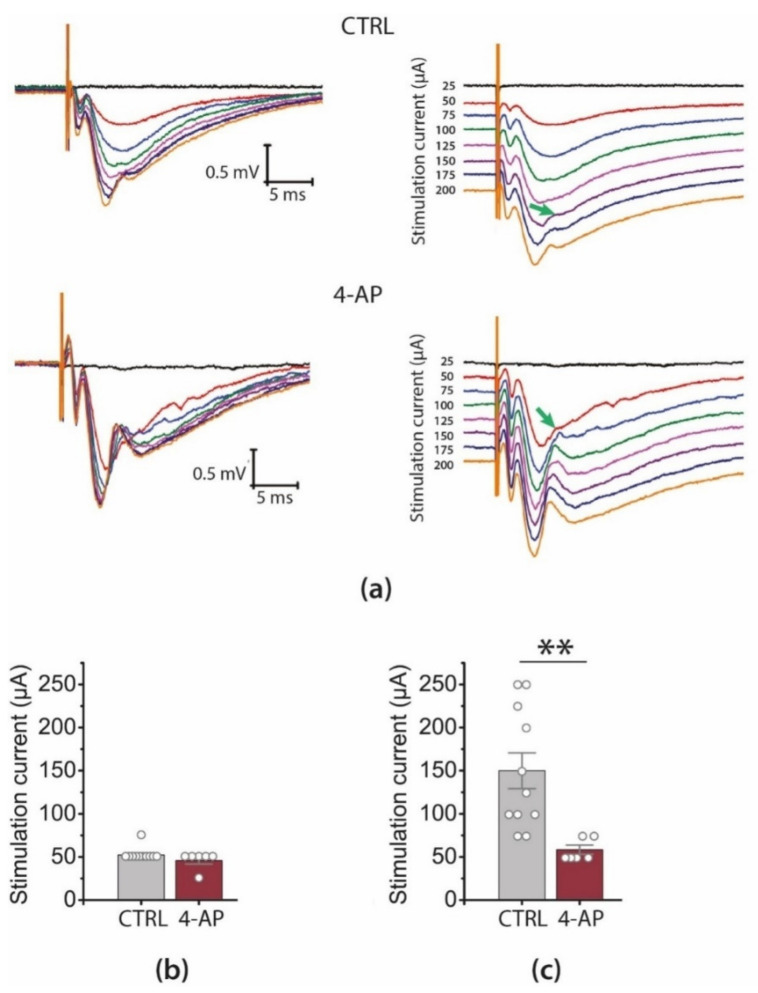
The effect of the short-term epileptiform activity on the basic synaptic transmission at CA3-CA1 hippocampal synapses. (**a**) Examples of local field excitatory postsynaptic potentials (fEPSP) recorded in stratum radiatum in control (CTRL) and after the period of the short-term epileptiform activity (4-AP). On the right, the same recordings are shown with the shift. The arrows point to the notches corresponding to the population spikes in the fEPSP recordings. Diagrams show the threshold of fEPSP initiation (**b**) and the threshold of population spike generation (**c**). All the data are presented as mean ± standard error of the mean, and each dot represents an individual value. ** *p* < 0.01: A significant difference versus the control group (*t*-test).

**Figure 3:**
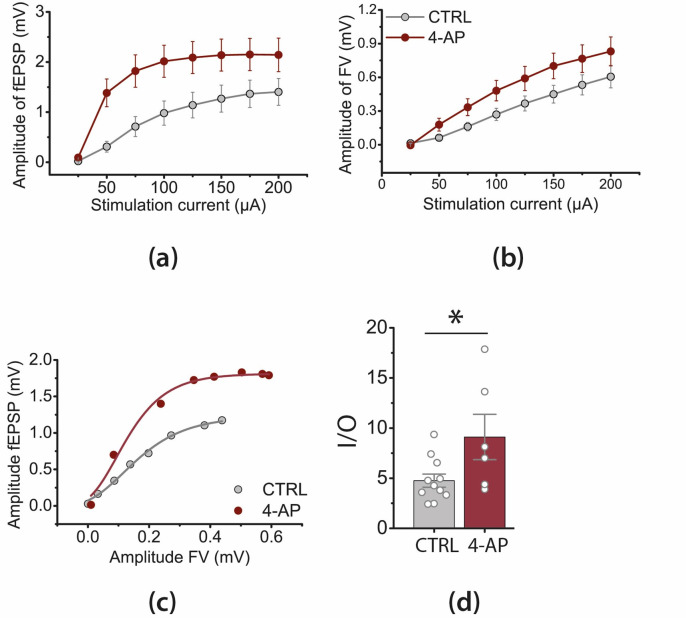
The effect of the short-term epileptiform activity on the basic synaptic transmission at CA3-CA1 hippocampal synapses. (**a**) Stimulation response relationships for fEPSP amplitudes, and (**b**) presynaptic fiber volley (FV), accordingly. (**c**,**d**) Changes in the maximal I/O slope after the period of the short-term epileptiform activity * *p* < 0.05, a significant difference versus the control group according to the Student’s *t*-test.

We registered fEPSPs in response to extracellular stimulation of Shaffer collaterals at a range of current intensities (Figure 2a). Even in one hour, the 4-AP-treated slices (4-AP slices) exhibited significantly increased excitability compared with the control. Although the threshold of fEPSP initiation was similar in both groups (Figure 2b, control: 52 ± 2 µA, *n* = 11; 4-AP–slices: 46 ± 4 µA; *n* = 6; *t*-test = 1.49, *p* = 0.16), the amplitude of fEPSP increased significantly faster with increasing stimulation current strength (Figure 3a, repeated-measures ANOVA: F_7,105_ = 4.3, *p* < 0.001). In addition, the threshold of population spike generation was much lower than that in the control slices (Figure 2c, control: 150 ± 21 µA, *n* = 11; 4-AP–slices: 58 ± 5 µA; *n* = 6; *t*-test = 3.18, *p* < 0.01).

To determine whether this increase in synaptic strength could result from enhanced presynaptic excitability, we have plotted the relationships between FV amplitude, a measure of presynaptic axon depolarization, and stimulus strength in control and 4-AP–slices (Figure 3b). We found that these relationships did not differ between the groups (effect of epileptiform activity: F_1,105_ = 3.4, *p* = 0.08), suggesting that the excitability of presynaptic axons was not affected.

To estimate the efficacy of basal synaptic transmission, we assessed the average slope of I/O curves plotted as fEPSP amplitudes vs. FV amplitudes (Figure 3c,d). Using a sigmoidal Gompertz function [17] to determine the maximum rise slope of the curves, we found that this value was significantly larger in 4-AP–slices (9 ± 2) than in control ones (4.8 ± 0.7, *t* = 2.35, *p* = 0.03; Figure 3d).

Together, these data suggest that short-term epileptiform activity increases neuronal excitability in the CA1 hippocampal area by increasing the synaptic efficacy in the CA3–CA1 synapses.

**Figure 4:**
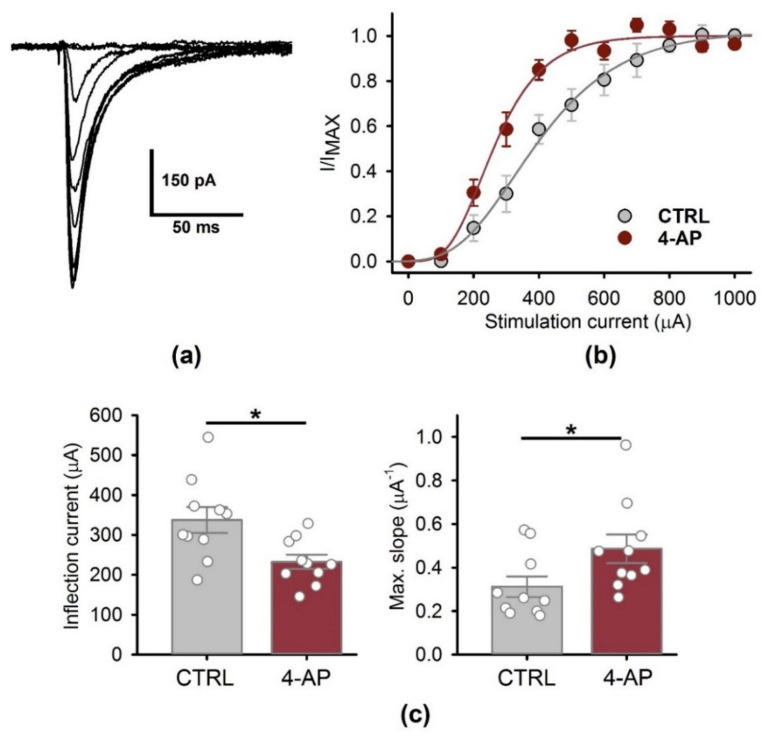
The effect of a period of epileptiform activity on the AMPAR-mediated eEPSC. (**a**) A representative set of 11 AMPAR-mediated eEPSCs, induced by the stimulation current of the increasing magnitude (from 0 to 1000 µA with the step of 100 µA). (**b**) The peak amplitude of AMPAR-mediated response vs. stimulus strength in control conditions and following a period of epileptiform activity (*n* = 10 for both cases). The data were normalized to the maximal response and fitted with the Gomperz function (Equation (1)). (**c**) The parameters of the Gompertz function under control conditions and following a period of epileptiform activity. A decrease in the inflection current (left) and an increase in the maximal slope of the curve (right) were detected; * *p* < 0.05, a significant difference versus the control group according to the Student’s *t*-test.

Next, we performed a similar experiment using the patch-clamp recording technique (Figure 4). We determined the relationships between the amplitude AMPAR-mediated eEPSCs and the stimulation current magnitude and then fitted them with the Gompertz function (Figure 4a,b).

In the presence of intact GABAergic transmission, we saw that following a period of epileptiform activity, the inflection current was decreased, and the slope of the curve was increased compared to the control (Figure 4c). These results indicate that smaller stimulation currents could evoke the same amplitude AMPAR-mediated eEPSCs (Figure 4).

Several factors can potentially contribute to the increased excitability of pyramidal neurons following epileptiform activity. This may be due to an increase in the probability of glutamate release, the number of receptors on the postsynaptic membrane, or the input resistance of the membrane. The latter would result in increased membrane depolarization for the same amount of incoming current through the synaptic receptors.

### 3.3. Biophysical Properties of CA1 Pyramidal Neurons

**Figure 5:**
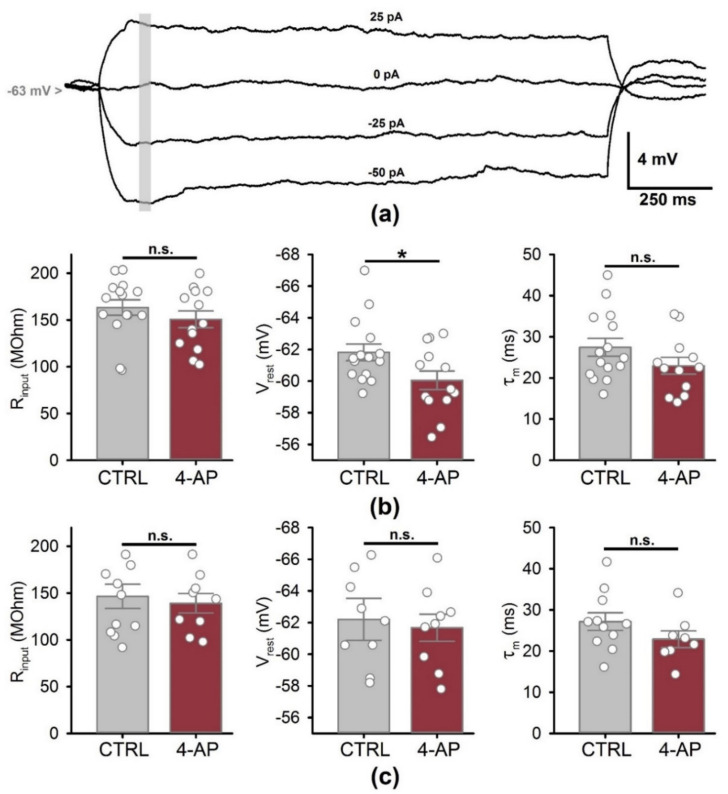
Changes of the subthreshold membrane properties of the neurons following a period of epileptiform activity induced by 4-aminopyridine-containing solution (4-AP). (**a**) A representative set of subthreshold responses to current steps from −50 to +25 pA. The gray bar indicates the time interval used to obtain the average values of membrane potential for the estimation of the input resistance. (**b**) Comparison of the membrane properties with the intact GABAergic synaptic transmission. (**c**) The exact comparisons in the presence of bicuculline, a GABAa receptor blocker. Data are presented as the mean with standard error of the mean. Each circle represents a value obtained in an individual neuron. * *p* < 0.05, a significant difference versus the control group according to the Student’s *t*-test.

The change in membrane properties may explain the fact that we observed a significant increase in the amplitude of fEPSPs, but saw more minor changes in postsynaptic currents. To estimate the effect of short-term epileptiform activity on biophysical properties of hippocampal neurons, we recorded the responses of CA1 pyramidal neurons to current steps (from −50 to +25 pA with an increment of 25 pA). We evaluated input resistance, resting membrane potential, and membrane time constant (Figure 5). With the intact inhibitory synaptic transmission, only a slight increase of the resting membrane potential from −61.8 ± 0.5 mV to −60.1 ± 0.5 mV was detected following a period of seizures (Figure 5b; *t*-test, *p* = 0.03), while the other two parameters were unaltered. No significant changes in any of these parameters were detected in the presence of bicuculline, a GABAa receptor blocker (Figure 5c). The observed depolarizing effect of GABAergic transmission may indicate that changes in the driving force of Cl^−^ ions that occur during epileptiform activity [24] persist for at least one hour. Thus, epileptiform activity had almost no effect on the subthreshold properties of the CA1 pyramidal neurons. 

**Figure 6:**
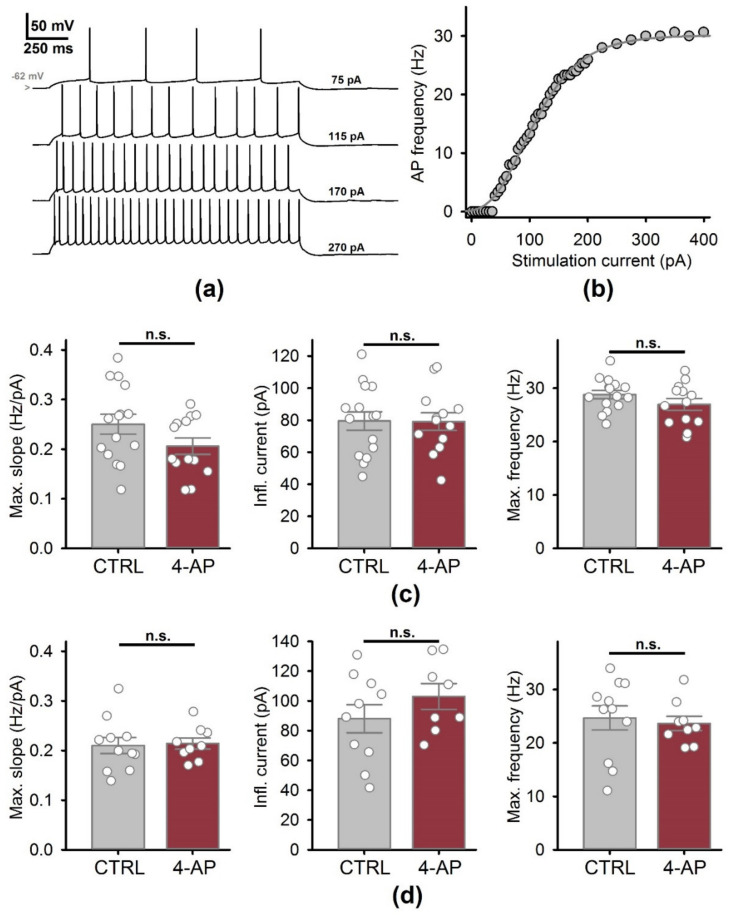
The firing properties of CA1 neurons do not change following a period of epileptiform activity induced by a 4-aminopyridine-containing solution (4-AP). (**a**) A representative set of voltage responses to depolarizing current steps was used to obtain the f/I curve (**b**). The data were fitted with the Gompertz function. (**c**) The comparisons of the parameters of the f/I curves, obtained with the intact GABAergic synaptic transmission. (**d**) Same comparisons in the presence of bicuculline, a GABAa receptor blocker. In both cases, no significant changes were detected (*t*-test was used for all comparisons).

Additionally, we investigated whether epileptiform activity affected the firing properties of hippocampal neurons (Figure 6). We fitted the rising parts of the f/I curve with the Gompertz equation (Equation (1)) and investigated whether the obtained parameters were altered after epileptiform activity (Figure 6a,b). We detected no significant changes in the maximal slope, inflection current, and the maximal frequency of the AP generation, both with intact GABAergic inhibition (Figure 6c) and in the presence of bicuculline (Figure 6d). Taken together, these results indicate that a period of epileptiform activity did not change the intrinsic excitability of CA1 neurons. 

### 3.4. Presynaptic Properties of CA1 Pyramidal Neurons 1 H after the Epileptiform Activity

To assess possible changes in the probability of glutamate release, we measured the frequency of mEPSCs and the paired-pulse ratio of eEPSCs. These parameters are traditionally employed to evaluate the transmitter release probability [32].

**Figure 7:**
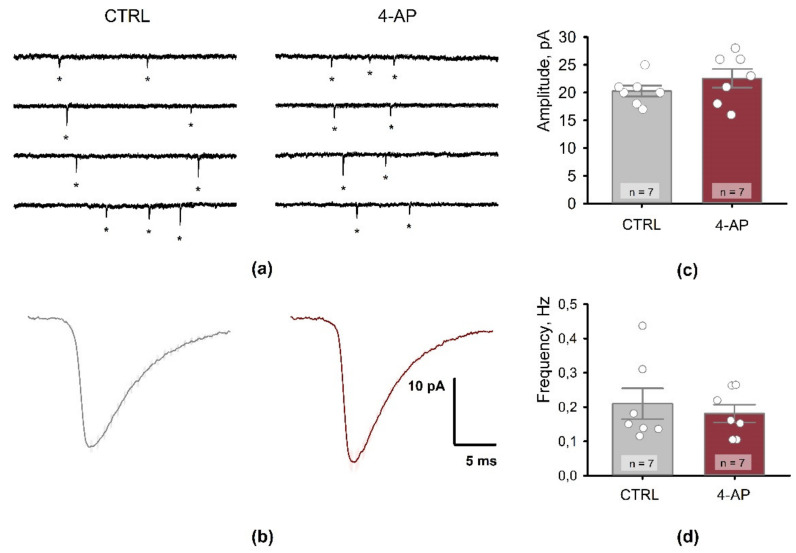
Properties of miniature excitatory postsynaptic currents (mEPSCs) 1 h after the period of short-term epileptiform activity. (**a**) Miniature EPSCs registered in the CA1 pyramidal neuron in the control (left) and 1 h after epileptiform activity (right). V_hold_ = −80 mV. (**b**) Representative examples of averaged mEPSCs from control (black) and 4-AP (red) pyramidal neurons. Graphs showing amplitude (**c**) and frequency (**d**) of mEPSCs in control (CTRL) and 1 h after epileptiform activity (4-AP). Data are presented as the mean with standard error of the mean. Each circle represents a value obtained in an individual neuron. No significant difference was detected between the control and 4-AP groups.

The registration of mEPSCs was carried out in the presence of tetrodotoxin (0.5 μM) and GABAa receptor blockers (picrotoxin, 50 μM and bicuculline, 10 μM; Figure 5a). Neither frequency (control: 0.21 ± 0.05 Hz; *n* = 7 vs. 4-AP–group: 0.18 ± 0.03 Hz; *n* = 7; *t*-test = 0.55, *p* = 0.60) nor amplitude (control: 20.3 ± 1.0 pA; *n* = 7 vs. 4-AP–group: 22.6 ± 1.7 pA; *n* = 7; *t*-test = 1.17, *p* = 0.26) differed significantly from control values (Figure 7).

**Figure 8:**
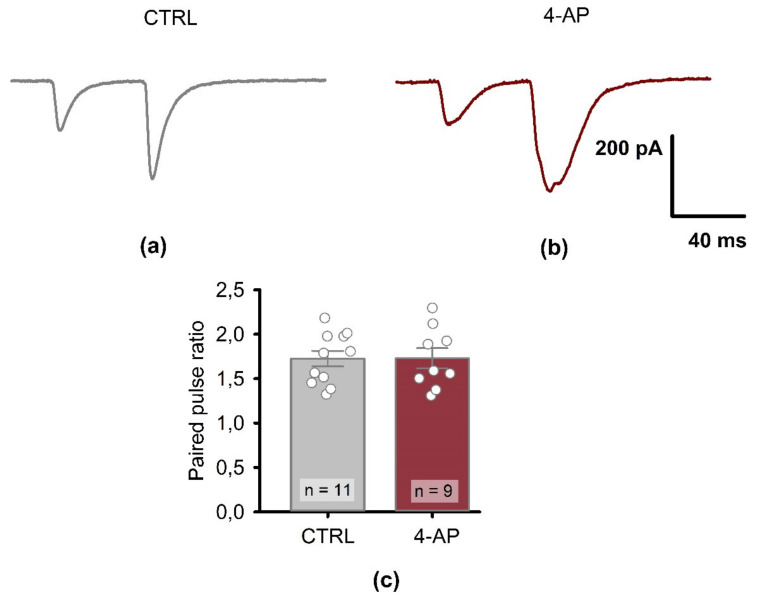
The paired-pulse amplitude ratio (PPR) of eEPSC recorded in the CA1 region of rat hippocampus in control and 1 h after short-term epileptiform activity. Representative examples of CA1 pyramidal neuron responses to a paired stimulus (inter-stimulus interval = 50 ms) in control (**a**) and 1 h after EA (**b**). (**c**) The bar graph shows PPR in control (CTRL) and 1 h after epileptiform activity (4-AP). Data are presented as the mean with standard error of the mean. Each circle represents a value obtained in an individual neuron. No significant difference was detected between the control and 4-AP groups.

The eEPSC responses to paired stimuli are shown in Figure 8. There was no significant change in PPR following epileptiform activity (control: 1.72 ± 0.09; *n* = 11 vs. 4-AP group: 1.73 ± 0.11; *n* = 9; *t*-test for independent samples = 0.02, *p* = 0.98).

The absence of differences in the frequency of mEPSCs and PPR indicates that the probability of glutamate release from presynaptic terminals has not changed.

### 3.5. Postsynaptic Properties of CA1 Pyramidal Neurons

**Figure 9:**
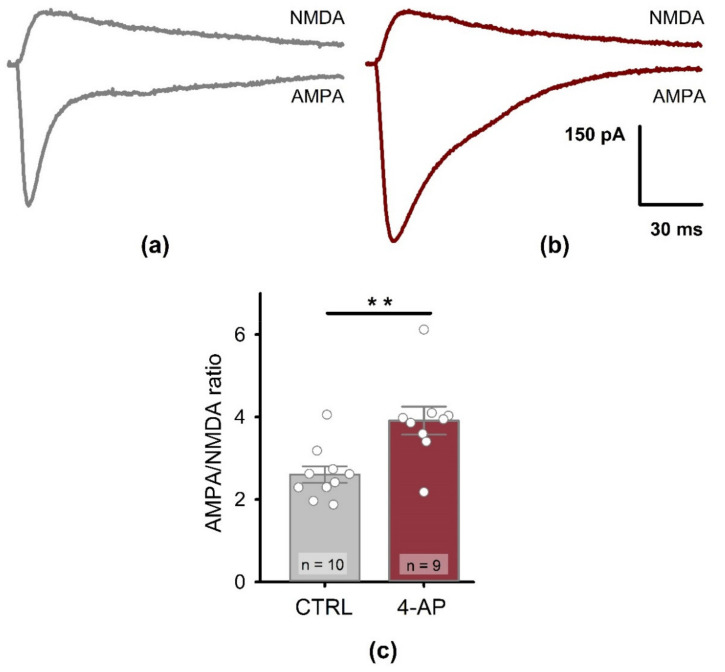
Postsynaptic properties of CA1 pyramidal neurons before and after the period of short-term epileptiform activity induced by 4-aminopyridine-containing solution (4-AP). Representative examples of AMPAR- or NMDAR-mediated currents in control (**a**) and 1 h after the period of short-term epileptiform activity induced by 4-AP (**b**). AMPAR-mediated responses were recorded −80 mV in the presence of bicuculline (20 μM), a GABAa receptor blocker. NMDAR-mediated EPSCs were recorded at +40 mV in the presence of bicuculline and DNQX (10 μM), an AMPAR antagonist. (**c**) The bar graph shows the AMPA/NMDA amplitude ratio increase after the period of the short-term epileptiform activity. Data are presented as the mean with standard error of the mean. Each circle represents a value obtained in an individual neuron. ** *p* < 0.01: A significant difference with the control group (*t*-test).

As shown above, in this model, epileptiform activity strongly enhances AMPAR-mediated neurotransmission. Therefore, we tested whether epileptiform activity alters the contribution of AMPAR- and NMDAR-mediated currents at the postsynaptic membrane. We found that epileptiform activity leads to a significant increase in the AMPA/NMDA ratio (Figure 9, control: 2.61 ± 0.20; *n* = 10 vs. 4-AP group: 3.91 ± 0.34; *n* = 9; *t*-test for independent samples = 3.39, *p* < 0.01). These results indicate the incorporation of new AMPARs into the postsynaptic membrane.

## 4. Discussion

A brief period of epileptiform activity increased hippocampal excitability, as demonstrated by the change in the I/O ratio of the fEPSPs. We tested several possible mechanisms, including changes in intrinsic membrane properties of neurons, and pre- and postsynaptic alterations. Neither input resistance nor other essential biophysical properties of hippocampal CA1 pyramidal neurons were affected by epileptiform activity. Furthermore, we did not detect any differences in the PPR of eEPSC amplitudes nor the frequency of mEPSC, leading us to conclude that 4-AP-induced epileptiform activity did not affect glutamate release probability. The absence of changes in the amplitude of fiber volley also indicates that presynaptic properties of glutamatergic transmission are not responsible for the observed increase in excitability. However, epileptiform activity in the 4-AP model increased the AMPA/NMDA ratio, suggesting that the alterations in the properties of postsynaptic glutamatergic receptors are the most likely explanation for the enhancement of basic synaptic transmission.

Our data are consistent with results obtained in other studies focused on the effects of short-term epileptiform activity in vitro. The 10-min perfusion of hippocampal slices with high K^+^ (10 mM) solution changed the slope of fEPSP recorded in the stratum radiatum of CA1. The observed potentiation reached its maximum level about 30 min after washout and was still detectable 60 min after washout [33]. Similar results were obtained in two other studies: (1) The potentiation of the fEPSPs was observed 50 min after the washout of high-K^+^-containing solution [34] and (2) at least 40 min after the washout of 4-AP (200 µM) [9]. Interestingly, even a brief period of epileptiform activity (40 s) has been observed to potentiate the amplitudes of fEPSPs (20 min after the washout), albeit hippocampal slices were exposed to very high levels of extracellular K^+^ (50 mM KCl) [18]. Organotypic hippocampal slice cultures demonstrated potentiation at CA3-CA1 synapses in response to a very brief period of epileptiform activity (0.5–3 min) induced either by bicuculline or by Mg^2+^-free solution. Potentiation of the fEPSP amplitude lasted at least 15 min after the washout of Mg^2+^-free solution and at least 30 min after the washout of bicuculline-containing solution [19]. In another study, using a high K^+^ model, the potentiation of the fEPSP slope in the CA1 area was reported, while the amplitude of FV was not affected [35].

The blockade of GABAaR-mediated inhibition and subsequent epileptiform activity has also been shown to lead to persisting changes in the properties of fEPSPs recorded in CA3 stratum radiatum/moleculare. Potentiation, defined as at least a 20% increase in the rising phase slope of fEPSPs, has been seen as long as 120 min after the washout of penicillin (2000 IU/mL) and cessation of spontaneous bursting [36]. 

Several mechanisms of increased excitability of hippocampal neural networks after epileptiform activity have been elucidated. For instance, a change in the neuronal network activity level can alter the intrinsic membrane properties of neurons [37,38,39]. Input resistance, especially, demonstrates close ties with mechanisms of homeostatic and nonhomeostatic plasticity [40]. It should be noted that 4-AP alters the intrinsic membrane properties of neurons by inhibiting voltage-gated potassium ion channels, expanding action potentials [41]. However, in our experiments, we measured the membrane properties of neurons as early as one hour after washout of 4-AP, so this effect of 4-AP can be neglected. 

In patients with pharmacoresistant epilepsy, neuronal loss in the CA1 region is frequently observed [42], suggesting that CA1 pyramidal neurons are among the most vulnerable cells to seizures. In this study, we found no significant effect of epileptiform activity on the passive membrane properties or firing properties of CA1 hippocampal neurons, although some studies have shown such changes. For example, 4-AP-induced epileptiform activity in the neocortex increased input resistance of parvalbumin-expressing neurons and reduced the action potential threshold for parvalbumin-expressing and pyramidal neurons both [43]. Apart from the 4-AP model, an increase in input resistance has been observed in the CA1 neurons of genetically epilepsy-prone rats [44], kindled rats [45], and in the pentylenetetrazole model [17]. However, after acute kainate-induced status epilepticus, there were no changes in input resistance in CA1 neurons [46], and the resting membrane potential and input resistance in piriform cortex neurons were not affected by abnormal activity induced by repeatedly applied tetanic stimulation [47]. In the lithium-pilocarpine model, seizures decreased the input resistance in entorhinal neurons [16]. As for other biophysical properties, there had been a decrease demonstrated in the membrane time constant in entorhinal and prefrontal neurons [16], significant membrane depolarization in CA1 neurons of kindled rats [45], latency of action potentials was prolonged, and the action potential half-width was increased 3–4 h after acute kainate-induced SE [46]. The exposure of immature hippocampal-cultured neurons to tetrodotoxin (0.5 μM) for 7–9 days, which led to spontaneous discharges, also affected the biophysical properties of cultured neurons. Neurons exhibited action potential broadening, lack of afterhyperpolarization, and had higher firing rates long after the medium was returned to standard composition [48]. The abnormal neuronal activity has also been shown to decrease A-type potassium currents [15] and hyperpolarization-activated currents [49,50], and increase T-type calcium channel-mediated currents [51,52] and persistent sodium currents [53]—the alternations mentioned above all have an impact on passive and active neuronal properties. Thus, changes in membrane properties depend primarily on the model used and the duration of epileptic activity. Likely, the short period of epileptiform activity in the model used was not sufficient to affect the membrane properties of neurons. Therefore, this mechanism is not involved in increasing the excitability of hippocampal neural networks.

In the presented work, we did not reveal presynaptic changes after epileptiform activity. The application of 4-AP affects the paired-pulse ratio, and during continuous perfusion of brain slices with 4-AP paired-pulse facilitation, turns into paired-pulse depression [9,54,55], suggesting an increase in neurotransmitter release probability. The frequency of mEPSCs was also significantly elevated during 4-AP-induced epileptiform activity, supporting that idea [9]. However, the duration of this effect remains unclear. In a model using overnight incubation in the bicuculline (50 µM), an increase in the frequency of mEPSC was detected. However, this is probably not due to changes in the probability of neurotransmitter release since no differences in the paired-pulse ratio were observed. Instead, this can be caused by the conversion of silent synapses into functional synapses [56].

We believe that postsynaptic changes are the primary mechanism of increased excitability of hippocampal neural networks. An increase in the AMPA/NMDA ratio is in favor of this assumption. It indicates that epileptiform activity led to the incorporation of AMPARs into the postsynaptic membrane. The incorporation of AMPARs is a well-known mechanism of synaptic plasticity that mediates activity-dependent synaptic changes during learning and memory [57]. The regulation of AMPAR trafficking to and from synapses involves lateral diffusion [58] and vesicular trafficking [59]. When on the membrane, AMPARs usually rapidly diffuse, while upon long-term potentiation (LTP), AMPARs get trapped at postsynaptic sites [58,60]. As confirmed recently, receptor trapping and clustering occur selectively opposite presynaptic release sites to ensure optimal receptor activation on neurotransmitter release [61,62]. 

AMPAR-mediated plasticity during epileptiform activity is rapid and often involves the incorporation of calcium-permeable AMPARs. The change in AMPAR-mediated transmission can be triggered by a 10–20-min period of epileptiform activity, as demonstrated in the in vitro epilepsy model [62] and pilocarpine model [63]. Peak AMPAR-mediated responses have been increased 2-fold during 4-AP-induced epileptiform activity in the entorhinal cortex, remained potentiated 15 min after short-term epileptiform activity in vitro, and this potentiation was shown to be NMDAR-dependent and at least partly mediated by the incorporation of calcium-permeable AMPARs [21]. There are multiple reports of changes in AMPAR protein expression levels and AMPAR subunit phosphorylation in the hippocampus several hours after seizures. The phosphorylation of 2 GluR1 subunit sites (S831 and S845) has been detectable 1 h after hypoxia-induced seizures and reached its maximum of 24 h after seizures [64]. Another study reports that surface expression of the GluA1 subunit was increased 60 min after the beginning of pilocarpine-induced SE [65]. A different pattern of AMPAR protein expression has been seen 3 h after pilocarpine-induced seizures. Reduced expression of GluA1, GluA3, and GluA4 subunits has been reported, in parallel with an elevation of GluA2 subunit expression [66]. 

Many studies also noted that epileptiform activity alters the AMPA/NMDA ratio and mEPSC in the hippocampus. In organotypic hippocampal slice cultures, an overnight incubation in the bicuculline (50 μM) increased the amplitude of mEPSCs and the AMPA/NMDA ratio. The addition of the NMDAR blocker CPP to the incubation solution prevented the changes in the AMPA/NMDA ratio and properties of miniature EPSC, pointing to the NMDAR-mediated nature of discussed changes [56]. Potentiation of AMPAR-mediated currents was noted 1 h after hypoxia-induced seizures in postnatal day 10 rats. Amplitudes of mEPSCs were elevated 1 h after seizures [64]. Amplitudes of mEPSCs recorded from CA1 pyramidal neurons were also increased in slices obtained from animals that had undergone pilocarpine-induced status epilepticus [65].

In our study, the amplitude of mEPSC remained indistinguishable from control levels. The difference in the effect of seizures on the amplitude of mEPSCs and eEPSCs in our experiments may arise because miniature events reflect the sheer broadness of the efferents CA1 receives from other areas. In contrast, the stimulation of Shaffer collaterals only provides CA3 input. Furthermore, it has been shown recently that spontaneous and synchronous transmitter release are distinct processes [67,68,69]. 

Overall, our data emphasize that AMPARs play a crucial role in seizure-induced synaptic plasticity. Considering that even a brief episode of epileptiform activity resulted in significant postsynaptic changes, the therapeutic strategies that rely on pharmacological modulation of postsynaptic glutamatergic receptors appear to have a good chance at alleviating damage associated with seizures or even preventing epileptogenesis.

## Data Availability

The data presented in this study are available on request from the corresponding author.
